# Interleukin-6 trans-signaling increases the expression of carcinoembryonic antigen-related cell adhesion molecules 5 and 6 in colorectal cancer cells

**DOI:** 10.1186/s12885-015-1950-1

**Published:** 2015-12-16

**Authors:** Reinhild Holmer, Georg H. Wätzig, Sanjay Tiwari, Stefan Rose-John, Holger Kalthoff

**Affiliations:** Division of Molecular Oncology, Institute for Experimental Cancer Research, University Hospital Schleswig-Holstein, 24105 Kiel, Germany; CONARIS Research Institute AG, Kiel, Germany; Section Biomedical Imaging, Department of Radiology and Neuroradiology, University Hospital Schleswig-Holstein, Kiel, Germany; Institute of Biochemistry, Christian-Albrechts-University, Kiel, Germany

**Keywords:** IL-6, Hyper-IL-6, Trans-signaling, CEA, Inflammation, Tumor-associated antigens, Tumor marker, Colon cancer, Colitis-associated cancer

## Abstract

**Background:**

Colorectal cancer (CRC) is among the five most frequent causes for cancer-related deaths in Europe. One of the most important tumor-associated antigens for CRC is carcinoembryonic antigen-related cell adhesion molecule 5 (CEACAM5), which is involved in cell adhesion, migration, anoikis, tumor invasion and metastasis. Its family member CEACAM6 is also upregulated in adenomas and carcinomas of the colon and an independent predictor of poor survival. Previous studies have reported a link between upregulation of CEACAM5 and interleukin-6 (IL-6). IL-6 plays an important role in CRC progression, and signaling is mediated via two pathways (classic and trans-signaling). However, this link could not be confirmed by other studies, and the role of IL-6 trans-signaling in the CEACAM5 upregulation has not been elucidated. Moreover, the impact of IL-6 on the expression of CEACAM6 has not yet been examined.

**Methods:**

The expression of IL-6, IL-6 receptor (IL-6R), glycoprotein (gp) 130, CEACAM5 and CEACAM6 was analyzed by RT-PCR, Western blot, flow cytometry or qPCR. Colon cell lines were incubated with IL-6 or Hyper-IL-6 (mediating IL-6 trans-signaling), and subsequently, the expression of CEACAMs was determined by qPCR or Western blot. FLLL31, an inhibitor of the phosphorylation of signal transducer and activator of transcription-3 (STAT3), was used to determine the role of STAT3 phosphorylation.

**Results:**

We confirmed that colon carcinoma cell lines express IL-6 and IL-6R. We observed only a weak upregulation of CEACAM5 and CEACAM6 by classic IL-6 signaling, but a strong increase by IL-6 trans-signaling. This upregulation depended on the phosphorylation of STAT3.

**Conclusions:**

Our data show the upregulation of the tumor-associated antigens CEACAM5/6 by trans-signaling of the pro-inflammatory cytokine IL-6. This mechanism may contribute to the tumor-promoting role of IL-6 and could therefore be a target for therapeutic intervention in particular by specific inhibitors such as sgp130Fc.

**Electronic supplementary material:**

The online version of this article (doi:10.1186/s12885-015-1950-1) contains supplementary material, which is available to authorized users.

## Background

CRC is still one of the leading causes of cancer deaths in Europe. According to calculations for the year 2014, it ranks second in men and third in women [1]. Several risk factors exist, including smoking, alcohol consumption, diabetes and inflammation [2, 3]. The link between inflammation and tumorigenesis is exemplified by patients with colitis-associated cancer (CAC). These are CRC patients that have previously suffered from inflammatory bowel disease (IBD). It is well-known that IBD patients have a higher risk of developing CAC/CRC [4, 5].

One of the key cytokines in IBD as well as in CRC is IL-6 [6]. IL-6 is a pleiotropic cytokine involved in various processes of innate and adaptive immunity [7, 8]. In the classic IL-6 signaling pathway, IL-6 binds to the membrane-bound IL-6R, which subsequently transmits the signal via the recruitment and homodimerization of two gp130 subunits. Consequently, an intracellular cascade is activated involving STAT3, mitogen-activated protein kinase (MAPK) and phosphatidylinositol-4,5-bisphosphate 3-kinase (PI3K) activation [9]. Whereas gp130 is ubiquitously expressed, IL-6R expression is restricted to only a few cell types, such as hepatocytes and certain leukocytes. However, a soluble form of IL-6R (sIL-6R) is generated by protease-mediated receptor shedding from the membrane or by alternative splicing. In contrast to some other soluble receptors, the sIL-6R does not act as an antagonist. Instead, it binds to IL-6 and trans-activates cells that only express gp130. This process was termed trans-signaling [9]. It is selectively inhibited by a naturally occuring soluble form of gp130 (sgp130). This knowledge was used to generate a potent and selective inhibitor of trans-signaling by fusing the sgp130 protein to the Fc part of a human IgG1 antibody. The resulting fusion protein is called sgp130Fc [9] and the optimized variant FE 999301 has already entered clinical development for the treatment of inflammatory bowel disease.

Several studies demonstrated a significant role of IL-6 in IBD as well as in CRC. These studies were recently reviewed by Waldner and Neurath, who concluded that IL-6 is the "master regulator of intestinal disease" [6]. Interestingly, in most studies, the pro-inflammatory and tumor-promoting activity of IL-6 was mediated via IL-6 trans-signaling [6, 10].

A causal link between IL-6 and CEACAM5 is revealed by significant association of serum levels of IL-6 with high serum levels of CEACAM5 [11, 12]. CEACAM5 (also called carcinoembryonic antigen, CEA) is one of the best-known tumor-associated antigens for CRC [13–15]. It is expressed in normal mucosal cells of the colon, but overexpressed in adenocarcinomas of the colon. In addition, its serum levels are elevated in CRC patients [15]. CEACAM5 is an adhesion molecule that was shown to be involved in cell adhesion, migration, anoikis, tumor invasion and metastasis [16, 17]. Furthermore, it activates inhibitory CEACAM1 signaling in natural killer cells (NK cells) and thereby blocks the cytotoxicity of NK cells [17]. CEACAM6, another family member of the carcinoembryonic antigen family, is already upregulated in benign precursor lesions like hyperplastic colorectal polyps and early adenomas [18]. Moreover, CEACAM6 is an independent predictor of poor survival for CRC patients [19] and is involved in tissue architecture and colonocyte differentiation [20].

IL-6 was previously shown to increase the expression of CEACAM5 on some CRC cells [21, 22]. However, this relationship was not observed for all CRC cell lines, and another study only found a very small and not significant stimulatory effect of IL-6 on the CEACAM5 expression [23]. To our knowledge, no study has yet examined the relationship between IL-6 trans-signaling and CEACAM5 and CEACAM6. Thus, the aim of this study was to systematically analyze the impact of IL-6 classic and trans-signaling on the expression of CEACAM5 and CEACAM6 in colorectal cancer cells.

## Methods

### Cell culture and proteins

The human colorectal adenocarcinoma cell lines HT29 (called HT29p for ’parental’ cells to distinguish it from other HT29 derivatives in our laboratory) and SW480 were obtained from the American Type Culture Collection (ATCC). HT29c cells had been generated in our laboratory by repeated injection of HT29 cells into the portal venous system of nude rats, subsequent isolation from liver metastases and reculturing in vitro [24, 25]. Colo357 cells, derived from a metastasis of a pancreatic adenocarcinoma, were a kind gift of Dr. R. Morgan (Denver, CO) [26]. These cells were routinely cultured in Roswell Park Memorial Institute (RPMI)-1640 medium (Gibco/Life Technologies, Darmstadt, Germany) supplemented with 10 % fetal bovine serum (FBS, PAN-Biotech, Aidenbach, Germany), 1 mM sodium pyruvate (Gibco) and 2 mM glutaMAX (Gibco). The human colorectal adenocarcinoma cell line Caco-2 was obtained from ATCC. It was cultured in Dulbecco’s Modified Eagle’s Medium (DMEM) (Gibco) supplemented with 10 % FBS, 1 mM sodium pyruvate and 2 mM glutaMAX. The normal mucosa-derived colon cell lines CSC1 [27] and NCM460 [28] were a kind gift of Dr. Mary Pat Moyer (San Antonio, TX, USA). These cell lines were maintained in M3 Base cell culture medium complete (M300A-500, Incell, San Antonio, TX, USA) with 10 % FBS. Ba/F3-gp130/IL-6R cells are Ba/F3 pre-B cells lacking endogenous gp130, which had been stably transfected with IL-6R and gp130 as a model system for IL-6 signaling [29, 30]. They were cultured in DMEM high glucose medium (Gibco) supplemented with 10 % FBS, 1 mM sodium pyruvate, 2 mM glutaMAX and 1 ng/ml IL-6. IL-6 and Hyper-IL-6 – a fusion protein of IL-6 and sIL-6R mimicking the IL-6 trans-signaling complex – were produced by the group of Prof. Stefan Rose-John as previously described [31, 32]. All cells were maintained at 37 °C in a humid atmosphere with 5 % CO2 and routinely checked for mycoplasma contamination with the MycoTrace kit (PAA/GE Healthcare, Cölbe, Germany).

### RNA isolation and cDNA synthesis

RNA was isolated using the RNeasy Plus Mini Kit (Qiagen, Hilden, Germany). RNA concentration was measured in a Nanodrop spectrophotometer (Thermo Fisher Scientific, Dreieich, Germany) and quality-checked on a 1 % agarose gel. 2 μg of RNA were reverse-transcribed into cDNA using the Maxima First Strand cDNA Synthesis Kit (Thermo Fisher Scientific).

### Reverse transcriptase polymerase chain reaction (RT-PCR)

PCR was performed using the Dream Taq Green Polymerase (Thermo Fisher Scientific). The primer sequences are depicted in Table 1, and the following conditions were used: initial denaturation: 95 °C, 2 min; denaturation: 95 °C, 30 s; annealing: 60 °C, 30 s; extension: 72 °C, 1 min (40 cycles); final extension: 72 °C, 10 min. The PCR product was analyzed by agarose gel electrophoresis on a 2 % agarose gel.

**Table 1:** Primers used for RT-PCR

Name	Sequence	Amplicon [bp] (mRNA/genomic)	Reference
hu_mIL6R_For	CATTGCCATTGTTCTGAGGTTC	280/not amplified	[54]
hu_IL6R_Rev	GTGCCACCCAGCCAGCTATC	278 (mIL6R), 280 (sIL6R)/not amplified	[54]
hu_gp130C	GGTACGAATGGCAGCATACA	713/10170	[55]
hu_gp130D	CTGGACTGGATTCATGCTGA	713/10170	[55]
hu_IL6_For1	TCCACAAGCGCCTTCGGTCC	621/not amplified	primerblast
hu_IL6_Rev1	TTGCCGAAGAGCCCTCAGGCT	621/not amplified	primerblast
hu_RPL22_For	TCGCTCACCTCCCTTTCTAA	250/6652	[56]
hu_RPL22_Rev	TCACGGTGATCTTGCTCTTG	250/6652	[56]

*RPL22* ribosomal protein L22

### Quantitative real-time polymerase chain reaction (qPCR)

cDNA was diluted 100-fold in nuclease-free water. 2 μl of diluted cDNA were used in a 20 μl reaction with FastSybr Green mastermix (Applied Biosystems/Life Technologies). The primer sequences are depicted in Table 2, and the following conditions were used: initial denaturation: 95 °C, 20 s; denaturation: 95 °C, 3 s; annealing/extension: 60 °C, 30 s (usually 40 cycles). Specificity of the product was verified by melt curve analysis and agarose gel electrophoresis.

**Table 2:** Primers used for qPCR

Name	Sequence 5’-3’	Amplicon [bp] (mRNA/genomic)	Reference
hu_CEA_For	CTTTATCGCCAAAATCACGC	138/6195	http://primerdepot.nci.nih.gov
hu_CEA_Rev	CCAGCTGAGAGACCAGGAGA	138/6195	http://primerdepot.nci.nih.gov
hu_CEACAM6_For1	GCATGTCCCCTGGAAGGA	179/1076	[57]
hu_CEACAM6_Rev1	CGCCTTTGTACCAGCTGTAA	179/1076	[57]
hu_RPL22_For	TCGCTCACCTCCCTTTCTAA	250/6652	[56]
hu_RPL22_Rev	TCACGGTGATCTTGCTCTTG	250/6652	[56]
hu_PPIC_For	GGAAAAGTCATTGATGGGATG	127/1907	sequence from Eva Simon, Kiel
hu_PPIC_Rev	CAAAAGGCGTTTTCACGTCTA	127/1907	sequence from Eva Simon, Kiel
hu_SDHA_For	ATTTGGTGGACAGAGCCTCA	126/not amplified	sequence from Eva Simon, Kiel
hu_SDHA_Rev	CTGGTATCATATCGCAGAGACCT	126/not amplified	sequence from Eva Simon, Kiel

*RPL22* ribosomal protein L22, *PPIC* peptidylprolyl isomerase C, *SDHA* succinate dehydrogenase complex, subunit A, flavoprotein

### Phorbol-12-myristate-13-acetate (PMA) stimulation and enzyme-linked immunosorbent assays (ELISAs)

For PMA stimulation, HT29p cells were seeded in a 96-well plate. On the next day, the medium was changed to remove non-adherent or dead cells. After 72 h, the supernatants were collected to measure the baseline (unstimulated) sIL-6R production of the cells (data not shown). Subsequently, the medium was changed, and the cells were stimulated for 2 h at room temperature (RT) with medium containing either 100 nM PMA (Calbiochem/Merck, Darmstadt, Germany) dissolved in dimethyl sulfoxide (DMSO) or 0.5 % DMSO as solvent control in triplicate wells. Supernatants from the triplicate wells were harvested and centrifuged for 15 min at 16,000 x g and 4 °C to remove cells and cellular debris. The purified supernatants were stored at −80 °C until ELISA analysis. sIL-6R concentrations were measured using ELISA kits (R&D Duoset, R&D Systems, Wiesbaden, Germany) according to the manufacturer’s instructions.

### Analysis of STAT3 phosphorylation and CEACAM expression by Western blotting

To analyze the phosphorylation of STAT3 and CEACAM5/6, HT29p cells were seeded in 6-well plates. After 48 h, the medium was replaced by serum-free medium. The next morning, cells were stimulated with different concentrations of IL-6 or Hyper-IL-6, a fusion protein of IL-6 and sIL-6R mimicking the IL-6 trans-signaling complex (see above). After 15 min (STAT3) or 48 h (CEACAM5/6), the cells were lysed with radioimmunoprecipitation assay (RIPA) buffer and stored at −20 °C until analysis for STAT3 phosphorylation in Western blots.

For Western blots, the lysates were thawed on ice, sonicated and centrifuged (13,000 rpm, 15 min, 4 °C) to remove cellular debris. Protein concentration was determined with the DC assay (Bio-Rad Laboratories, Munich, Germany). Equal amounts of protein were loaded onto a 4–20 % tris-glycine gel (Life Technologies) and separated by SDS-PAGE. Proteins were blotted on a PVDF membrane (Immobilon-FL; Millipore/Merck, Darmstadt, Germany), blocked with 5 % milk or bovine serum albumin for 1 h at RT and incubated with the primary antibody overnight at 4 °C. Secondary antibody incubation was performed for 1 h at RT. All washes were performed with TBS supplemented with 0.01 % Tween-20. Blots were dried with methanol and scanned in an Odyssey imager (LI-COR, Bad Homburg, Germany). Alternatively, horseradish peroxidase (HRP)-coupled secondary antibodies were used. After incubation, these membranes were incubated with a substrate for electrochemiluminescence (ECL), and readout was performed using films (Amersham Hyperfilm ECL, both from GE Healthcare, Munich, Germany) and an Agfa Curix 60 developing machine (Agfa, Mortsel, Belgium). The following antibodies were used: P-STAT3 (#9131, Cell Signaling Technology/New England Biolabs, Schwalbach, Germany), STAT3 (#9139, Cell Signaling Technology), β-actin (ab6276, Abcam, Cambridge, UK), goat-anti-mouse-IRDye680 (LI-COR), goat-anti-rabbit-IRDye800CW (LI-COR), CEACAM5 (T84.66, kindly provided by Stefanie Nittka, Mannheim, Germany), CEACAM6 (AM02001PU-N, Acris, Herford, Germany), goat-anti-rabbit IgG-HRP (#7074, Cell Signaling) and horse-anti-mouse IgG-HRP (#7076, Cell Signaling).

### CEACAM analysis by flow cytometry

For the analysis of CEACAM molecules by flow cytometry, cells were harvested after Accutase treatment (GE Healthcare, Munich, Germany). Subsequently, all steps were performed on ice. Cells were washed with FACS buffer (PBS with 2 % human serum, 2 mM EDTA and 0.02 % sodium azide), blocked with FACS buffer for 15 min and stained with primary antibodies diluted in FACS buffer (30 min on ice). Subsequently, they were washed three times with FACS buffer and incubated with the fluorochrome-coupled secondary antibody. After washing, the cells were incubated with FACS buffer containing 7AAD (BD Bioscience, Franklin Lakes, NJ, USA). Afterwards, the cells were measured in a FACScalibur (BD). Weasel software v3.0 (chromocyte, Sheffield, UK) was used for data analysis. Dead cells were excluded by gating for the 7AAD-negative cells, as dead cells were previously shown to be false positive for CEACAM5/6. The following antibodies were used: CEACAM5 (C1P83, produced by our group as previously described [33, 34]), CEACAM6 (AM02001PU-N, Acris, Herford, Germany), mouse IgG1-isotype control (X0931, Dako, Glostrup, Denmark) and anti-mouse-IgG-Alexa488 (Invitrogen/Life Technologies, Darmstadt, Germany).

### Ethics statement

Our study is in compliance with the Helsinki Declaration. We did not perform a clinical trial or used any materials from clinical specimens and therefore, no consent of patients was necessary. Only the cell lines used in this work were obtained from the sources indicated in the “Cell culture and protein” section.

## Results

### Colon cell lines express molecules mediating IL-6 signaling

**Fig. 1:**
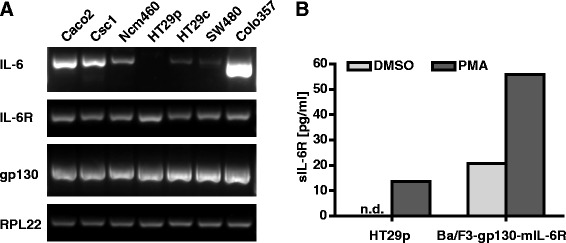
Colon cells express IL-6, IL-6R and gp130. **a**, RNA from different colorectal cancer cells (Caco-2, HT29p, HT29c, SW480) and normal mucosa-derived colon cells (CSC1, NCM460) was extracted, reverse-transcribed into cDNA and analyzed for the expression of IL-6, IL-6R and gp130. The pancreatic cell line Colo357 served as a positive control. Ribosomal protein L22 (RPL22) was used as a reference gene to monitor equal transcription of cDNA. **b**, HT29p cells were seeded in 6-well plates. After 72 h, the medium was replaced by medium containing either the solvent dimethyl sulfoxide (DMSO) or phorbol-12-myristate-13-acetate (PMA) in DMSO. Supernatants were collected after 2 h, and the sIL-6R concentration was determined by ELISA. Ba/F3-gp130-mIL-6R cells were used as a positive control. n.d. not detected

To understand the impact of IL-6 on colon cells, we first analyzed by reverse transcription polymerase chain reaction (RT-PCR) whether mucosa-derived colon cell lines (CSC1, NCM460) and colorectal cancer cell lines (Caco-2, HT29p, HT29c, SW480) express key components of the IL-6 signaling pathway. The mucosa-derived cell lines were originally isolated from histologically normal colonic margins from patients undergoing resection for colon adenocarcinomas and represent the disease in its early stages of transformation [27, 35]. Most cell lines expressed IL-6 mRNA. However, the level among the cell lines varied, and the parental HT29 cell line (HT29p) did not show any IL-6 expression (Fig. 1a). In contrast, the cell line HT29c, which was derived from HT29p cells using a successive intrahepatic selection procedure in rats [24, 25], expresses IL-6 (Fig. 1a). Moreover, all cell lines showed mRNA expression of the IL-6R and of the co-receptor gp130.

However, we did not detect the IL-6R on the surface of colorectal cancer cells by flow cytometry (data not shown). Therefore, we assumed that either the expression level was low or that the receptor was shed. IL-6R is mainly shed by ADAM17, which is strongly activated by phorbol-12-myristate-13-acetate (PMA) [36]. Stimulation of HT29p cells with PMA led to an increase of the sIL-6R concentration in the supernatant (Fig. 1b), demonstrating that colorectal cancer cells express the IL-6R protein on their membrane, and that it can be shed from the surface.

### CEACAM5 and CEACAM6 are expressed in most colon cell lines

**Fig. 2:**
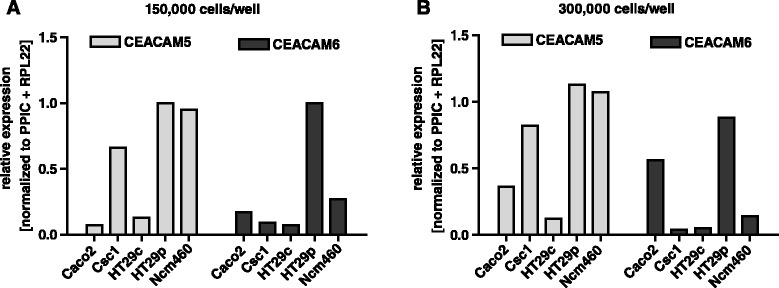
Most colon cell lines express mRNA of CEACAM5 and CEACAM6. **a**, 150,000 or 300,000 (**b**) colorectal cancer cells (Caco-2, HT29p, HT29c, SW480) or normal mucosa-derived colon cell lines (CSC1, NCM460) were seeded in 6-well plates. After 48 h, the RNA was extracted and analyzed by qPCR for the expression of CEACAM5 and CEACAM6. PPIC and RPL22 were used as reference genes. SW480 cells did not show any expression (not shown)

Before we analyzed the relationship between IL-6 and CEACAMs, we examined the expression of CEACAM5 and CEACAM6 in the normal mucosa-derived and colorectal cancer cell lines. Most of these cell lines expressed CEACAM5 as well as CEACAM6. However, the level varied between different cell lines. On the mRNA level, HT29p and NCM460 cells showed the highest expression of CEACAM5 (Fig. 2). HT29p also had the highest mRNA level of CEACAM6. Interestingly, the variant HT29c showed a much lower expression of CEACAM5 and CEACAM6 than HT29p. The cell line Caco-2 clearly increased its expression of CEACAM5 and CEACAM6 with higher confluency (Fig. 2). SW480 cells did not express any CEACAM5/6 (data not shown).

**Fig. 3:**
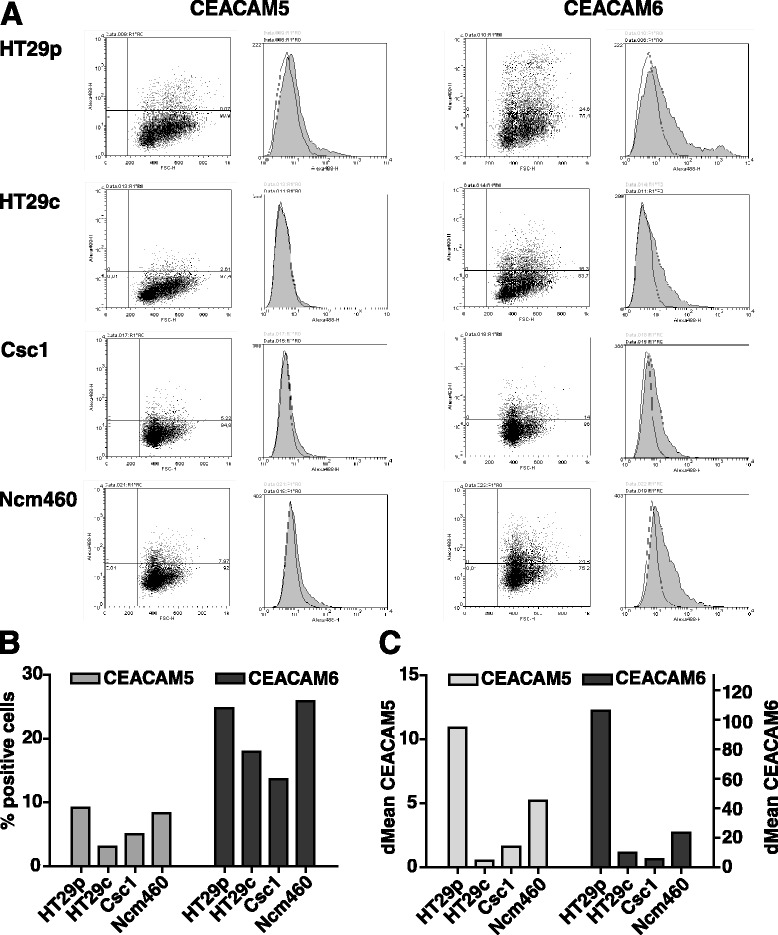
Most colon cell lines express CEACAM5 and CEACAM6 proteins on their surface. **a**, The CEACAM5/6 expression of different colorectal cancer cell lines (HT29p, HT29c) and normal mucosa-derived colon cell lines (CSC1, NCM460) was analyzed by flow cytometry using specific antibodies (grey) or an unspecific isotype control (white). The amount of positive cells (**b**) as well as the difference in the mean fluorescence intensity (dMean) between the specific staining and the isotype control (**c**) was analyzed

On the protein level, we analyzed the CEACAM5/6 surface expression by flow cytometry (Fig. 3). In a given cell population, only a fraction of cells was positive for CEACAM5 and CEACAM6. Similar to our findings on the mRNA level, HT29p cells had the highest surface protein expression level (represented by the difference in the mean fluorescence intensity between isotype control-stained and CEACAM5/6-stained cells), as well as the highest percentage of positive cells (Fig. 3). NCM460 cells showed a similar amount of positive cells, but the expression level was lower than in HT29p cells. Again, HT29c cells exhibited a lower expression of CEACAM5/6 than HT29p, and SW480 did not show any surface expression.

### IL-6 trans-signaling upregulates the expression of CEACAM5 and CEACAM6 in colon cancer cells

**Fig. 4:**
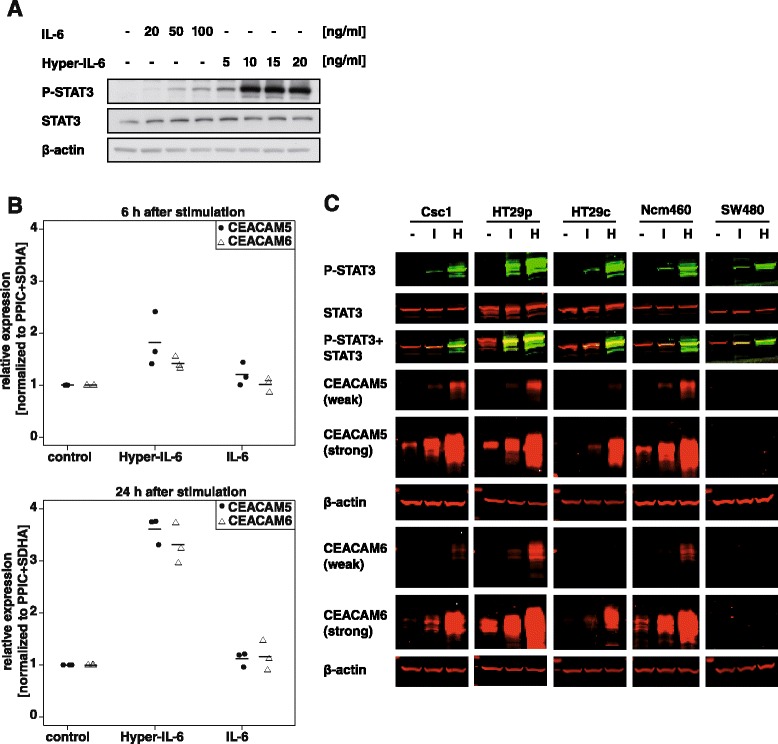
Hyper-IL-6 strongly activates STAT3 and increases the expression of CEACAM5 and CEACAM6. **a**, HT29p cells were seeded in 6-well plates, serum-starved overnight and stimulated with different concentrations of IL-6 or Hyper-IL-6. After 15 min, the cells were lysed and the lysates analyzed for STAT3 phosphorylation in Western blots by ECL. **b**, HT29p cells were seeded in 6-well plates, serum-starved overnight and stimulated with IL-6 (100 ng/ml) or Hyper-IL-6 (15 ng/ml). After 6 or 24 h, the RNA was isolated and analyzed by qPCR for the expression of CEACAM5 and CEACAM6. PPIC and SDHA were used as reference genes. Three independent experiments are shown. **c**, Different cell lines were seeded in 6-well plates, serum-starved overnight and treated with IL-6 (100 ng/ml; “I”) or Hyper-IL-6 (15 ng/ml; “H”). After 15 min (to examine STAT3 phosphorylation) or 48 h (CEACAM expression), cells were lysed and the lysates analyzed for the phosphorylation of STAT3 and the expression of CEACAM in Western blots and scanned in the Odyssey near-infrared imaging system. β-actin was used to monitor equal protein loading. For CEACAM5/6, the blots are shown in different intensities

To study the effect of IL-6 classic and trans-signaling, cells were stimulated either with IL-6 or Hyper-IL-6 (consisting of human IL-6 linked by a flexible peptide chain to the soluble form of the IL-6 receptor) at different concentrations. For this preliminary experiment, the cell line HT29p was chosen, because it did not show an endogenous IL-6 expression (Fig. 1a). As one of the earliest steps in the IL-6 signaling cascade, we analyzed the phosphorylation of STAT3. While IL-6 only weakly activated STAT3, Hyper-IL-6 led to a strong phosphorylation even at low concentrations (Fig. 4a). This suggests that HT29p cells express IL-6R only in small amounts and are not very responsive to IL-6.

To answer the question whether IL-6 signaling leads to an upregulation of the CEACAM5/6 expression, we treated HT29p cells with IL-6 (100 ng/ml) or Hyper-IL-6 (15 ng/ml) and analyzed the CEACAM5/6 expression on the mRNA level after 6 and 24 h. After 6 h, only slight changes were observed, but after 24 h, expression of CEACAM5 and CEACAM6 was clearly increased by Hyper-IL-6 stimulation (Fig. 4b). In contrast, IL-6 only led to slight changes in the CEACAM5/6 expression (Fig. 4b).

We confirmed this finding at the protein level in the two normal mucosa-derived cell lines (CSC1, NCM460) and two representative colorectal cancer cell lines which express CEACAM5 and CEACAM6 (HT29p, HT29c) (Fig. 4c). STAT3 was strongly phosphorylated by Hyper-IL-6 in all of these cell lines. Stimulation with IL-6 led to a much weaker STAT3 phosphorylation, although its concentration was much higher than that of Hyper-IL-6. Consequently, the CEACAM expression was not as strongly increased as with Hyper-IL-6. The cell line SW480 did not express any detectable CEACAM5 or CEACAM6. This also did not change after treatment with IL-6 or Hyper-IL-6, indicating that CEACAM expression is not inducible by IL-6 *de novo*, but is typically stimulated by IL-6 trans-signaling (Fig. 4c). While the relationship between IL-6 and CEACAM5 expression was not clear in the literature, our data suggest that IL-6 leads to a small increase in the expression of CEACAM5 and CEACAM6, but that this increase is much stronger when IL-6 trans-signaling occurs. This may be due to the low IL-6R expression. Furthermore, we show here for the first time that CEACAM6 is upregulated by IL-6 trans-signaling.

### The phosphorylation of STAT3 is necessary for the Hyper-IL-6-mediated increase in CEACAM5/6

**Fig. 5:**
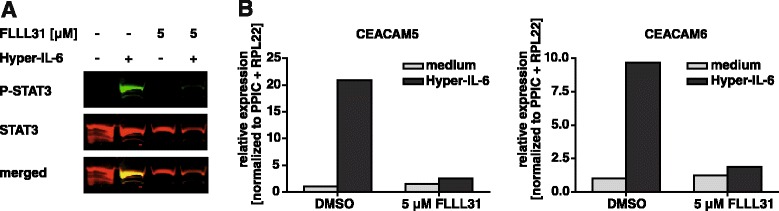
Inhibition of STAT3 phosphorylation prevents the Hyper-IL-6-mediated increase of CEACAM expression. **a**, HT29p cells were seeded in 6-well plates, serum-starved overnight and pre-treated with FLLL31 (5 μM) for 2 h. Subsequently, cells were treated with serum-free medium with or without Hyper-IL-6 (15 ng/ml). After 15 min, cells were lysed and the lysates analyzed for the phosphorylation of STAT3 in Western blots. **b**, After 24 h, RNA was isolated and analyzed by qPCR for the expression of CEACAM5 and CEACAM6. PPIC and RPL22 were used as reference genes

To analyze whether the Hyper-IL-6-mediated increase in CEACAM5/6 expression depends on the phosphorylation of STAT3, HT29p cells were pre-treated with FLLL31, a small molecule STAT3 inhibitor derived from curcumin [37]. Subsequently, the cells were stimulated either with normal serum-free medium or with Hyper-IL-6. FLLL31 clearly inhibited the Hyper-IL-6-induced phosphorylation of STAT3 (Fig. 5a) and the increase in CEACAM5/6 expression (Fig. 5b).

### IL-6 trans-signaling stabilizes hypoxia-inducible factor 1α (HIF-1α), and chemical stabilization of HIF-1α upregulates CEACAM5/6

IL-6 was shown to increase the expression of HIF-1α at the protein synthesis level [38]. HIF-1α, in turn, was described to upregulate CEACAM5 and CEACAM6 [39, 40]. Therefore, we tested whether the observed (Hyper-)IL-6-mediated CEACAM5/6 increase could be due to an increased HIF-1α level.

**Fig. 6:**
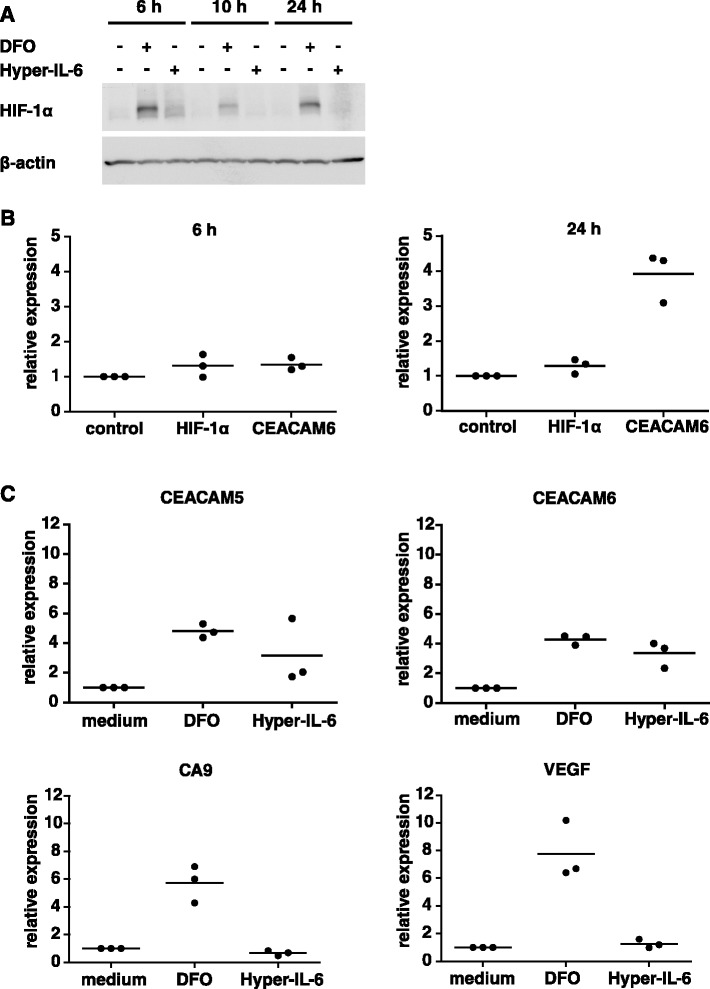
Hyper-IL-6 increases the expression of HIF-1α on the protein level. **a**, HT29p cells were treated with Hyper-IL-6 (15 ng/ml) or with 100 μM of deferoxamine mesylate (DFO) as a positive control. Cells were lysed at the indicated time points and analyzed by Western blotting for the expression of HIF-1α. **b**, HT29p cells were treated with Hyper-IL-6 (15 ng/ml). After 6 or 24 h, RNA was extracted and analyzed by qPCR for the expression of HIF-1α. The expression of CEACAM6 was used as a positive control. Three independent experiments are shown. **c**, HT29p cells were seeded in 6-well plates. Cells were serum-starved and subsequently stimulated with DFO (100 μM) or with Hyper-IL-6 (15 ng/ml). After 24 h, RNA was extracted and analyzed by qPCR for the expression of CA9, VEGF, CEACAM5 and CEACAM6. Three independent experiments are shown

We confirmed that treatment of HT29p cells with Hyper-IL-6 indeed increased HIF-1α on the protein level after 6 h of incubation. At later time points, this difference decreased (Fig. 6a). On the mRNA level, we did not detect a significant upregulation of HIF-1α mRNA (Fig. 6b). HIF-1α is chemically stabilized by the iron chelator deferoxamine mesylate (DFO). DFO inhibits prolyl hydroxylases, which degrade HIF-1α [41]. We used DFO as a control to confirm that HIF-1α leads to an upregulation of CEACAMs. Treatment of HT29p cells with DFO led to a clear upregulation of CEACAM5 as well as of CEACAM6 (Fig. 6c). The classic HIF-1α target genes CA9 and VEGF were used as positive controls. However, they were only upregulated by DFO but not by Hyper-IL-6, although Hyper-IL-6 increased the HIF-1α protein level in these cells. In summary, these data support the notion that HIF-1α plays a role in the Hyper-IL-6-induced CEACAM5/6 upregulation, but further studies are necessary to understand the complex mechanism of regulation.

## Discussion

In this study, we show that IL-6 trans-signaling significantly upregulates the expression of CEACAM5 and CEACAM6 in CRC cells. IL-6 trans-signaling is known to be important for the development of CRC [6]. We show that some CRC cells constitutively express IL-6, whereas all of the tested cell lines express IL-6R and gp130 on the mRNA level. However, cells only weakly responded to stimulation with IL-6. In comparison, IL-6 trans-signaling (induced by Hyper-IL-6 mimicking the IL-6/sIL-6R complex) strongly phosphorylated STAT3 and led to a significant increase in CEACAM5 and CEACAM6 expression. Interestingly, cell lines originally derived from normal mucosa [28] also expressed IL-6, IL-6R and gp130. This is consistent with other studies, demonstrating expression of IL-6 and mIL-6R in intestinal epithelial cells [42].

IL-6 classic signaling weakly phosphorylated STAT3 and increased the expression of CEACAM5 and CEACAM6 in different colon cell lines. In comparison, IL-6 trans-signaling had a much stronger effect. This may be due to a low IL-6R expression on the cell surface. The differential IL-6R expression could also be the explanation why some previous studies described an effect of IL-6 on the CEACAM expression [21, 22] while others did not [23]. Accordingly, we also observed a STAT3 phosphorylation and a CEACAM5/6 upregulation by classic signaling in the pancreatic cell line Colo357, which obviously expressed sufficient amounts of IL-6R (Additional file 1: Figure S1).

In HT29p cells, the observed influence of IL-6 trans-signaling on the CEACAM expression depended on the phosphorylation of STAT3, as an inhibitor of the STAT3 phosphorylation blocked the Hyper-IL-6-mediated CEACAM increase. Moreover, Hyper-IL-6 led to an increase in HIF-1α levels. Interestingly, this increase was only observed on the protein level but not on mRNA level, an effect previously described for IL-6. Briggs showed in his doctoral thesis that IL-6 increases the rate of HIF-1α synthesis (translation) rather than the rate of transcription [43], and STAT3 was shown to inhibit the degradation of HIF-1α [44]. Increased translation seems to be a common mechanism for HIF-1α increase after stimulation with growth/oncogenic stimuli [44–46].

Stabilization of HIF-1α by the hypoxia mimetic deferoxamine mesylate (DFO) led to an upregulation of CEACAM5 and CEACAM6. This suggested that HIF-1α might be involved in the Hyper-IL-6-mediated CEACAM5/6 upregulation. Moreover, STAT3 and HIF-1α had previously been shown to interact in transcriptional complexes to regulate the expression of HIF-1α target genes [47–50]. However, the classical HIF-1α target genes CA9 and VEGF were not upregulated by Hyper-IL-6 in our settings. Therefore, further studies are necessary to elucidate the detailed mechanism of transcriptional regulation of CEACAM5 and CEACAM6.

## Conclusions

In summary, we show in this study that IL-6 trans-signaling increases the expression of CEACAM5 and CEACAM6 in colon cells. This may be important for tumorigenesis, as CEACAM5 and CEACAM6 are involved in adhesion, migration, invasion and metastasis [17]. This study provides further support for inhibiting IL-6 trans-signaling as a clinical therapeutic strategy for colorectal cancer [51–53].

## Additional file

Additional file 1: Figure S1. IL-6 influences the expression of CEACAM5 and CEACAM6 in the pancreatic cancer cell line Colo357. Colo357 cells were treated with IL-6 (100 ng/ml) in serum-free medium or with the anti-IL-6R- antibody tocilizumab (10 or 100 μg/ml) in serum-containing medium to block endogenous IL-6 signaling for 24 h. RNA was isolated and qPCR performed to analyze the expression of CEACAM5/6. (PDF 95 kb)

## Abbreviations

BSA: bovine serum albumin
CEA: carcinoembryonic antigen
CEACAM: carcinoembryonic antigen-related cell adhesion molecule
CAC: colitis-associated cancer
CRC: colorectal cancer
DFO: deferoxamine mesylate
DMSO: dimethyl sulfoxide
ECL: electrochemiluminescence
ELISA: enzyme-linked immunosorbent assay
FBS: fetal bovine serum
gp130: glycoprotein 130
HIF-1α: hypoxia-inducible factor 1α
HRP: horseradish peroxidase
IBD: inflammatory bowel disease
IL-6: interleukin-6
IL-6R: IL-6 receptor
MAPK: mitogen-activated protein kinase
mIL-6R: membrane-bound form of the IL-6R
NK cells: natural killer cells
PBS: phosphate-buffered saline
PI3K: phosphatidylinositol-4,5-bisphosphate 3-kinase
PMA: phorbol-12-myristate-13-acetate
PPIC: peptidylprolyl isomerase C
qPCR: quantitative PCR
RPL22: ribosomal protein L22
RT-PCR: reverse transcriptase polymerase chain reaction
RIPA: radioimmunoprecipitation assay
RT: room temperature
SDHA: succinate dehydrogenase complex subunit A flavoprotein
sIL-6R: soluble IL-6 receptor
STAT3: signal transducer and activator of transcription-3
TBS: tris-buffered saline
