# Supplementary figures and images for: Interleukin-6 trans-signaling increases the expression of carcinoembryonic antigen-related cell adhesion molecules 5 and 6 in colorectal cancer cells
Source: BMC Cancer. 2015 Dec 16;15:975. doi: 10.1186/s12885-015-1950-1 (PMC4682226; doi:10.1186/s12885-015-1950-1)

**CEACAM5**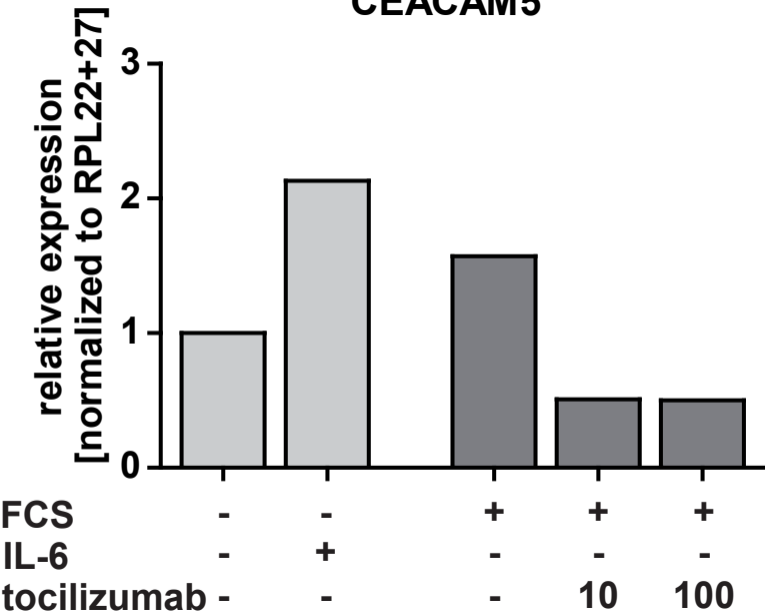**CEACAM6**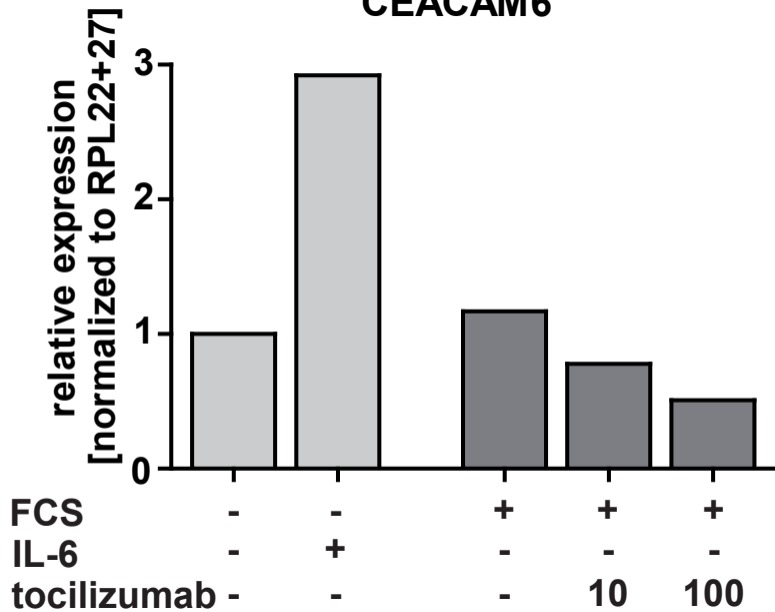

Supplement: Additional file 1: Figure S1. — IL-6 influences the expression of CEACAM5 and CEACAM6 in the pancreatic cancer cell line Colo357. Colo357 cells were treated with IL-6 (100 ng/ml) in serum-free medium or with the anti-IL-6R- antibody tocilizumab (10 or 100 μg/ml) in serum-containing medium to block endogenous IL-6 signaling for 24 h. RNA was isolated and qPCR performed to analyze the expression of CEACAM5/6. (PDF 95 kb) [file 12885_2015_1950_MOESM1_ESM.pdf]
